# LINC00441 promotes cervical cancer progression by modulating miR-450b-5p/RAB10 axis

**DOI:** 10.1186/s12935-020-01400-x

**Published:** 2020-08-04

**Authors:** Haiyan Han, Qingchun Shao, Xuejie Liu

**Affiliations:** 1 Reproductive Center, Affiliated Hospital of Weifang Medical College, No. 2428 Yuhe Road, Kuiwen District, Weifang, 261031 Shandong China; 2 Obstetrical Department 1, Affiliated Hospital of Weifang Medical College, No. 2428 Yuhe Road, Kuiwen District, Weifang, 261031 Shandong China

**Keywords:** LINC00441, Cervical cancer, miR-450b-5p, RAB10

## Abstract

**Background:**

As one of the most common gynaecological malignant tumors, cervical cancer (CC) has become an important public health issue. Emerging evidence has revealed long non-coding RNAs (lncRNAs) are crucial regulators of biological functions in cancers, including CC. And the oncogenic role of LINC00441 has been verified in hepatocellular carcinoma (HCC). But the molecular mechanism and biological functions of LINC00441 in CC remain unknown.

**Methods:**

qRT-PCR analysis detected the expression of genes in CC tissues or cells. CCK-8, colony formation, flow cytometry, transwell, western blot assays as well as animal studies were conducted to analyze the function of LINC00441 in CC. Luciferase reporter, RIP and RNA pull down assays were applied to verify the binding relations among the indicated genes.

**Results:**

LINC00441 was upregulated in CC tissues and cells. Further, LINC00441 depletion repressed cell proliferation and motility in vitro as well as tumor growth in vivo. LINC00441 could sponge miR-450b-5p to upregulate RAB10 expression. Finally, miR-450b-5p inhibitor or RAB10 upregulation counteracted LINC00441 knockdown-mediated function on the development of CC.

**Conclusions:**

LINC00441 drives CC progression by targeting miR-450b-5p/RAB10 axis, which might provide new idea for researching CC-related molecular mechanism.

## Background

Cervical cancer (CC), second to breast cancer, is the most deadly cancer among female cancer patients, with high morbidity and mortality [1]. As reported by the latest literature, it is estimated that the global age-standardized incidence of CC was 13.1 per 100,000 women, and the incidence varies greatly from country to country, ranging from 2 to 75 cases per 100,000 women [2]. Although great progress has made in CC screening and prevention of CC, such as Mitomycin C (MMC), a DNA alkylating agent, has been extensively utilized as a component of combination therapy [3], CC is still difficult to completely cure. It is reported that one-third of women treated will relapse and almost inevitably lead to death [4]. Current situation promotes us to identify effective biomarkers for diagnosis or prognosis of CC.

Long non-coding RNAs (lncRNAs) are emerging as a class of important regulators in cancers, which contains at least 200 nucleotides in length and lacks protein-coding capability [5]. LncRNA is involved in gene expression modulation and pathway activation, powerfully evidenced by the prevalent ceRNA network mechanism. For example, lncRNA SNHG1 activates β catenin/Wnt pathway by the sequestration of miR-302, miR-372, miR-373 and miR-520 in invasive pituitary tumors [6]. LncRNA EGOT drives gastric cancer tumorigenesis via Hedgehog signaling [7]. For CC, the widespread application of next-generation sequencing technologies contributes to identification of more and more lncRNAs [8]. For instance, lncRNA TCONS–_00026907 modulates CC progression by suppressing miR-143-5p [9]. LncRNA GAS5 attenuates CC carcinogenesis by modulating miR-196a and miR-205 [10]. LncRNA MEG3 is downregulated in CC and inhibits cell proliferation by modulating miR-21 [11]. RB1 divergent (also called LINC00441) is a newly-identified lncRNA whose expression is upregulated in hepatocellular carcinoma and gastric cancer and promotes their progressions [12, 13]. Through qRT-PCR analysis, we confirmed that LINC00441 was an abnormally upregulated lncRNA in CC patients’ tissues. Nevertheless, the function of LINC00441 in CC hasn’t been illustrated yet.

Further, the microRNAs (miRNAs) are also implicated in cancer progression. For illustration, inhibition of miR-203 obstructs cell growth and stemness in breast cancer via targeting SOCS3 [14]. Besides, miR-450b-5p inhibition mediated KIF26B activation contributes to the development of hepatocellular carcinoma by stimulating PI3K/AKT pathway [15]. MiR-450b-5p represses stemness and chemoresistance in colorectal cancer via targeting SOX2 [16]. And miR-450b-5p was predicted as the potential downstream miRNA of LINC00441. Furtherly, the miRNAs and lncRNAs have demonstrated to be implicated in tumorigenesis [17]. LncRNA NEAT1 facilitates CC cell growth through sponging miR-9-5p [18]. LncRNA ANRIL contributes to the development of CC by serving as a sponge of miR-186 [19]. In this research, we presumed whether LINC00441 affected CC progression via interacting with the miR-450b-5p.

Moreover, messenger RNA (mRNA), a component of ceRNA network, usually plays a decisive role in modulating cancer development, such as HMGA2 in hepatocellular carcinoma [20], MTDH in non-small cell lung cancer [21], and PAX8 in pancreatic cancer [22]. In our studies, RAB10 was found to be as the potential target gene of miR-450b-5p, and the assumption that LINC00441 promoted the development of CC via sequestering miR-450b-5p to upregulate RAB10 expression was worthy of confirming.

In this research, we elucidated that LINC00441 promoted CC progression by modulating miR-450b-5p/RAB10 axis, which might provide a potential therapeutic target for treatment of CC.

## Methods

### Tissue samples

Fifty-eight pairs of cervical cancer tissue samples and adjacent normal tissue samples were collected from June 2014 to July 2019, under the approval of the Ethics Committee of Affiliated Hospital of Weifang Medical College. The participants were all signed the informed consents and none had treated with chemotherapy or radiotherapy before surgery. Samples were sharply frozen in liquid nitrogen right after surgery and then stored at − 80 °C for subsequent analysis.

### Cell lines

Normal cervical cell line (Ect1/E6E7) and CC cell lines (HeLa, CaSki, SiHa and C33A) were procured from American Type Culture Collection (ATCC; Manassas, VA) and routinely cultured in Dulbecco’s Modified Eagle Medium (DMEM; Invitrogen, Carlsbad, CA) at 37 °C in 95% air and 5% CO_2_. 10% fetal bovine serum (FBS, Invitrogen, Carlsbad CA, USA) and 1% penicillin/streptavidin (Invitrogen) acted as the medium supplements. Culture medium was replaced every 3 days. Staurosporine was acquired from Sigma-Aldrich.

### Quantitative real-time polymerase chain reaction (qRT-PCR)

RNA sample was extracted by TRIzol reagent (Invitrogen) and then RNA quality was determined via NanoDrop spectrophotometer ND-8000 (NanoDrop Technologies; Thermo Fisher). Afterwards, RNA samples were converted into cDNA by use of Reverse Transcription Kit (Toyobo, Osaka, Japan). The relative expression of genes was measured by qPCR via SYBR Green Taq Mix (Takara) on ABI Prism 7900HT (Applied Biosystems, Foster City, CA), followed by calculation with 2^−∆∆Ct^ method. PCR process was as below: pre-denaturation at 95 ℃ for 10 min, 40 cycles of denaturation at 95 ℃ for 15 s, annealing at 60 ℃ for 1 min and extension at 72 ℃ for 30 s. All experimental results were standardized to the level of GAPDH or U6. Related primer sequences were provided in Table 1.

**Table 1:** List of primer sequences

**Primer sequences (5’-3’)**	
**LINC00441** (LINC00441-201; RB1-DT-201)	
Forward	TTGCAAAGTCGGCCAAAAC
Reverse	GCAGTCTGGACACTTGGTAC
**RAB10**	
Forward	TTCACACCATCACAACCTCC
Reverse	CCAGTAACATTCTTTCCACATCTTC
**NECAP2**	
Forward	GAAAACCTCCACCCTGATCC
Reverse	GTAAAGTCTCCCCAGATGTCAG
**CREG1**	
Forward	CGTGCCCTATTTCTACCTGAG
Reverse	GGGACTTTGTGGATCAAATCC
**CAPRIN1**	
Forward	TGAGTGGACAGTTGAAACGG
Reverse	CCATAAGGTCTTGTACTCGCTG
**CS**	
Forward	CATTGACTCTAACCTGGACTGG
Reverse	ACTTACATTGCCACCCTCATG
**PABPC3**	
Forward	GCAAATGTTAGGTGAACGGC
Reverse	CCAGTGATTTTCCCAGCAAG
**EIF5**	
Forward	GCCAAAGAGATTCGTGTCAAAG
Reverse	TCTCAACTTTCGGTACACTGG
**CASC4**	
Forward	TCAAGCAATCTCCCATCTTCC
Reverse	TTAAACGCTGAAGAGGACTGG
**GNS**	
Forward	CCATAGACCCAGAGCTTTTAGG
Reverse	TTGCTGAACATGAGACGGG
**TNPO1**	
Forward	CTAATGCCTCCACTGATCCAG
Reverse	GATACACAGGTTCACAGTACGG
**RHOBTB3**	
Forward	GCAGTCTGGACACTTGGTAC
Reverse	TCCTGATTCCCGTTTATGGTG
**PKD2**	
Forward	AGCGAGCCAAACTGAAGAG
Reverse	ATTCCCAGCGTTCCAACTC
**MTPN**	
Forward	TGGGCAGCTTGAAATCCTG
Reverse	TCAGCACCCTTTGACAGAA
**PHF14**	
Forward	TTGTGCCCTGTATGTTCCTG
Reverse	TGCAAACCCCAGTTCTAGC
**ASAP1**	
Forward	TCTATCCCCAAATGTGCAGTC
Reverse	GTCTTCACTCGCCTCACTTT
**UBE2V2**	
Forward	AGCCTGAAAGTAGAATGTGGAC
Reverse	TTGCCATTTTGCTAACACTGG
**HNRNPK**	
Forward	GTCGTGGCTCATATGGTGATC
Reverse	CAATTTTGATCGAAGCTCCCG
**GAPDH**	
Forward	CTCTGCTCCTCCTGTTCGAC
Reverse	GCGCCCAATACGACCAAATC
**U6**	
Forward	CTCGCTTCGGCAGCACA
Reverse	AACGCTTCACGAATTTGCGT
**miR-450b-5p** (uuuugcaauauguuccugaaua)	
Real time PCR:	
Forward: ACACTCCAGCTGGGTTTTGCAATATGTTCC	
Reverse transcription PCR:	
CTCAACTGGTGTCGTGGAGTCGGCAATTCAGTTGAGTATTCAGG	
**miR-744-5p** (ugcggggcuagggcuaacagca)	
Real time PCR:	
Forward: ACACTCCAGCTGGGTGCGGGGCTAGGGCTA	
Reverse transcription PCR:	
CTCAACTGGTGTCGTGGAGTCGGCAATTCAGTTGAGTGCTGTTA	
**Universal reverse** TGGTGTCGTGGAGTCG	

### Transfection

Cells of HeLa or CaSki at about 80% confluence were cultured in the 6-well plates for 48 h of transfection by use of Lipofectamine 2000 (Invitrogen). The sh-LINC00441#1/2/3 and the control sh-NC, miR-450b-5p mimics and the control miR-NC, sh-RAB10#1/2/3 and the control sh-NC, pcDNA3/1/LINC00441 and the empty vector control, miR-450b-5p inhibitor and the control miR-NC, pcDNA3.1/RAB10 and the empty vector control, were all acquired commercially from RiboBio (Guangzhou, China). And the transfection efficiency was approximately 80%. Related interference or overexpression sequences were provided in Table 2.

**Table 2:** List of interference/overexpression sequences

**Overexpression or interference sequence for miR-450b-5p (5’-3’)**	
**NC mimics**	GUCUCUUUAGUUAUUACAUAGA
**miR-450b-5p mimics**	UUUUGCAAUAUGUUCCUGAAUA
**NC inhibitor**	GAAAUAGACAUUCAAUACUAGU
**miR-450b-5p inhibitor**	UAUUCAGGAACAUAUUGCAAAA
**Interference sequence (5’-3’)**	
**sh-NC**	
CCGGGGGACCAGAGTACTGAGTCAACTCGAGTTGACTCAGTACTCTGGTCCCTTTTTG	
**sh-LINC00441#1**	
CCGGGAGCAGCTGAGCAAGGTCTAACTCGAGTTAGACCTTGCTCAGCTGCTCTTTTTG	
**sh-LINC00441#2**	
CCGGGCCCATCTGAACTCGTCAACTCTCGAGAGTTGACGAGTTCAGATGGGCTTTTTG	
**sh-LINC00441#3**	
CCGGGGAGCGCTGATAGACACTGTTCTCGAGAACAGTGTCTATCAGCGCTCCTTTTTG	
**sh-RAB10#1**	
CCGGAGAAGATCAAGCTACAGATATCTCGAGATATCTGTAGCTTGATCTTCTTTTTTTG	
**sh-RAB10#2**	
CCGGGCCTTCAATACTACCTTTATTCTCGAGAATAAAGGTAGTATTGAAGGCTTTTT	
**sh-RAB10#3**	
CCGGCAAGTGTGATATGGACGACAACTCGAGTTGTCGTCCATATCACACTTGTTTTT	
**Overexpression sequences (5’-3’)**	
**LINC00441**	
CTGGGGCGCTGGTCGGTGCGCGGGCTGGGACGCTAAGTCATGAGGAATTAAACTGGGAAACCTGGCGTGGGTCCAGGCGCCTTCCAGGAGGCATCCGGCGCGGCCCGGACGTGCTTCTACCCAGAACCACCCCCTCCAGGCCGGGGTGCACTTAACGGGGCTATACAAAGAGTCTGGTGGGTGACTGTGGGCCTCATCCCTATCCCGGGGTCTGATAGGGAAGACTCTCGGGCCCCGCAGGGAATATCTGGCTAGTGCACCCTCGGCGGGAGCGCCACTCTCCCTCCTTGGGCGGCGCCCCCCACCTCTCATCCCGCCCAATCCGTTTTGCAAAGTCGGCCAAAACAAAAACAAACTTGGAGCGCTGATAGACACTGTTATTGCCCATCTGAACTCGTCAACTGAGAAGGAAAGAGATGGTTTATGCTGTGAATAAAATACAGTTGTGCTGTGATGTAAATCCCCTCATCCTGGAGCAGCTGAGCAAGGTCTAAGGCAGGAAGAACCTTCAAGCAGGTCATGGCTTGCAGGCTCAACTCTGCCTGGCAGTGGCAAGGGCCAGGGCCAGTGACTGAAGCAACAAACTGCCAGATGACTGCAGCTCAAGCCAATGCACAAAGCCACTGCAACAAGAGAGGAAATTCTTCCATTTCCAAGTGTGTCGATTTCTCCCCATCAAGGAAAACAGCTAAGGCAATGTGTTGATATTTTCTTTGACCAAGTCATCAGGAGTCACATAGACATACAGCACTCAAGAAGTGGCTGTAACTAAAGGCACATCTCGTTCATCTACAACCCTTGTAATCCCAACCTTTGTCTGATGCACAAACCCTTCCCTAGTTCTCCCCACCACCACCACTGCACCTCCAAGTGGGGACAACGAATTGTTTCATTTGCATGTGGTTTTTCTCATGAAAAATATCATTAACATGAGACAGCCCCAAACCAAGGAAACCAGAAGTGGGGTTTGTGCTTGGTCTTACTGAGAGCTTTCCCTACTTGACCAGACCCCAGCATTGTTCTAGATTTGTCTCTTGTGGGCTACAGGAATGCAAACATTTTGTAGTTTCCTCTTTTTTGTTTTCTTAATGGCTTGATGAGCCACACATGTTTCTCTAAATGCATGATTTTAAATTCACACAA	
**RAB10**	
GTCTCAGGCAGCGCGCACGCCGGCGTGAGAGGGCACGGGGAAAAGGTGGCTCTGGCCGGGGTGGCTCGGTTTCCTGGGGCTATGTAACTGAGCTCGTCGACTTAGGGGTCCTTCTTCGCTGCCCTCGCCGCGTGCTAGCAGGGAGTTTCCGCTCGGGAGAGAGACTGTCCTCACGCCCGCTGCGCCTCCTCGACGGCAGAGCAGGCTTGCTCGCCCGTGGGAGCGTCCCGGCCGAGAAGCCCTGAGGGGGGAGGGGAGGCCATTTTGTCCCGACCGACTCCCCGGAACCGGGCGGACGGGCTGGGAGAGGCTGCGGAGCCGCGGTCGCCGCCCTCGGAGGCACTGGACGCCGCCACTGTCGGGGCTTCCTCAAAGCTGTTCGTAGGTCGCCCGCGCCGTCTCGAGCCTTTTTCCCACGCTTCCCCGGTCCTCCGGCCTGAGAACGCCCGAGTGAGGAGTTGGCCGTAGTGAGAGGGACCGATCCCTTGGGGCCGCCGGCGGCGAGAGCCCGAGCCGCTCCTCCCAATGGCGAAGAAGACGTACGACCTGCTTTTCAAGCTGCTCCTGATCGGGGATTCCGGAGTGGGGAAGACCTGCGTCCTTTTTCGTTTTTCGGATGATGCCTTCAATACTACCTTTATTTCCACCATAGGAATAGACTTCAAGATCAAAACAGTTGAATTACAAGGAAAGAAGATCAAGCTACAGATATGGGATACAGCAGGCCAGGAGCGATTTCACACCATCACAACCTCCTACTACAGAGGCGCAATGGGTATCATGCTAGTATATGACATCACCAATGGTAAAAGTTTTGAAAACATCAGCAAATGGCTTAGAAACATAGATGAGCATGCCAATGAAGATGTGGAAAGAATGTTACTAGGAAACAAGTGTGATATGGACGACAAAAGAGTTGTACCTAAAGGAAAAGGAGAACAGATTGCAAGGGAGCATGGTATTAGGTTTTTTGAGACTAGTGCAAAAGCAAATATAAACATCGAAAAGGCGTTCCTCACGTTAGCTGAAGATATCCTTCGAAAGACCCCTGTAAAAGAGCCCAACAGTGAAAATGTAGATATCAGCAGTGGAGGAGGCGTGACAGGCTGGAAGAGCAAATGCTGCTGAGCATTCTCCTGTTCCATCAGTTGCCATCCACTACCCCGTTTTCTCTTCTTGCTGCAAAATAAACCACTCTGTCCATTTTTAACTCTAAACAGATATTTTTGTTTCTCATCTTAACTATCCAAGCCACCTATTTTATTTGTTCTTTCATCTGTGACTGCTTGCTGACTTTATCATAATTTTCTTCAAACAAAAAAATGTATAGAAAAATCATGTCTGTGAGTTCATTTTTAAATGTACTTGCTCAGCTCAACTGCATTTCAGTTGTATTATAGTCCAGTTCTTATCAACATTAAAACCTATAGCAATCATTTCAAATCTATTCTGCAAATTGTATAAGAATAAAGTTAGAATTAACAATTTTATTTTGTACAACAGTGGAATTTTCTGTCATGGATAATGTGCTTGAGTCCCTATAATCTATAGACATGTGATAGCAAAAGAAACAAACAAAAGCCAGGAAAACACTCATTTTCGCCTTGAATATGTAAATGGGATTAATTTTGTCCTGTGCCTTATGTGGAAAGGAACTTCTTTGGTTTTCCTTTTTTGTTCTGGTGGAAGCATGTGCAGGAGACATATCATCCAAACATAAACCATTAAAATGTTTGTGGTTTGCTTGGCTGTAATTTTCAAAGTAGTTAATTGAGGACAAAGGGTAATGCAGAAGTGATAGCTTTGGTTTGCTGAGTCTTGTTTTAAGTGGCCTTGATATTTAAAACTATTCCTGCCACCATTTCTTCTCCTTGGCCACTTCTTCCTTGCGTCTCCCTGCATGCTGCTTTATTTGCTTCTCCCTCCCCAACCACCTCATGGTATATTTAAGAGTGAAAGGGACAAACTAGTAGGTTTGTCAAGTTTAATATAAAGCACTGATGTAACTTGCTAGGTAAACGGAAAGATAAGTTCTAACTGCCTACTATCCAATGTCAGTTAATTGGTGTCTTCCCCCCTCATTTGCTCTCTTCCCTAAAATGTGTCCCAGATGCCTTCATTTGCTGTTTTACTTCTATGTTCTGCTTTTCCTCCTCTCTTTGTTCCCTTCCTGTCTATCCATTGAGTTTATGAAATGGAAGAGTTAACTGCATGCACTAGTGTTTGGAGGGTGTTGTGGTTTGTCTTTCTAATTAGGTGTATAGCCTATTCACTTTCCTAGAATAAATCTCTTAACCTAAATTTGAGTAGTCTGCATTTTGGCAACTCCTCTAGCAGCTTGGTAGCCTAGTACAGGTTGTTTTTTTAAAAAAGGAAAAGCAGGAAGGAGGAGTGAATTTTATTAACATGTTTGCCAAATGTATTGAGATTTGGCCTCTGAAGAACACTTTTTCAGTGTTAAGTTTCTTTACCTTAAGATTCAGAAATACTTTAGAATATTATTAATTTTAAGTCCTGTCTTTACATCCTTTTGGAAAACTTGTATTACCATGGGTTTGGAAAAAGGACAACGAAAGGCTTTTCATGTAAAGATAAGATCTTTAGCTATCTCTAACCCTGTCCTTTTTTCACTGCATTTTTTCTAGTTTTGCTTCATTGCTTATCATTAGGATAGGGTAAGTGAAGTTTGCTATGCTGCTAGCATCCTAAGATGATACCTTTGTTGAAAGAATTGTGAATAGCATGATTCATTTCTAGCAGAGGCTGAGTTTAGGACAGCAGCTTCCATTGAGAAGTCTTTCTGTGTCGTGAATAGCATTTTAATGACCTCTTGGCTCACATAAGCAAACAACATAGGGACGTATCTGCTATGAAAATCCACAAATTTTTCAGATAGTGCCCTAAAAACAATTTTATATGCCTCACTGGTTGTTATTCTTAGGTTATTCCCACACTTGACTTTATCATTGTTTACTACTAGTAAAAAGCAGCATTGCCAAATAATCCCTAATTTTCCACTAAAAATATAATGAAATGATGTTAAGCTTTTTGAAAAGTTTAGGTTAAACCTACTGTTGTTAGATTAATGTATTTGTTGCTTCCCTTTATCTGGAATGTGGCATTAGCTTTTTTATTTTAACCCTCTTTAATTCTTATTCAATTCCATGACTTAAGGTTGGAGAGCTAAACACTGGGATTTTTGGATAACAGACTGACAGTTTTGCATAATTATAATCGGCATTGTACATAGAAAGGATATGGCTACCTTTTGTTAAATCTGCACTTTCTAAATATCAAAAAAGGGAAATGAAGTATAAATCAATTTTTGTATAATCTGTTTGAAACATGAGTTTTATTTGCTTAATATTAGGGCTTTGCCCCTTTTCTGTAAGTCTCTTGGGATCCTGTGTAGAAGCTGTTCTCATTAAACACCAAACAGTTAAGTCCATTCTCTGGTACTAGCTACAAATTCGGTTTCATATTCTACTTAACAATTTAAATAAACTGAAATATTTCTAGATGGTCTACTTCTGTTCATATAAAAACAAAACTTGATTTCCA	

### Cell-Counting Kit 8 (CCK-8) assay

The transfected CC cells (5 × 10^3^) were harvested and planted into 96-well plates. After incubated with indicated times (1, 2, 3, 4 days), cells were processed with 10 μL of CCK8 reagent (Dojindo, Osaka, Japan) for 2 h, following the standard method. The absorbance at 450 nm was finally examined at indicated time points by microplate reader (Thermo Fisher Scientific, Waltham, MA, USA).

### Colony formation assay

Clonogenic cells of HeLa or CaSki (1 × 10^3^ cells per well) were collected after 48 h of transfection and cultivated in 6-well culture plates for 14 days at the temperature of 37 ℃ in 5% CO2. Thereafter, cells were rinsed by PBS, fixated via 4% formaldehyde for 30 min, and stained with 0.5% crystal violet for 5 min. The colonies with over 50 cells was imaged and counted manually.

### Flow cytometry analysis

Cell apoptosis was monitored by use of flow cytometer (BD Biosciences, Franklin Lakes, NJ). After transfection, CC cells (1 × 10^6^) were harvested and mixed with the Annexin V-labelled with 7AAD and PE in 1 × Binding Buffer for 15 min at 37 ℃ in the dark. At length, cells were analyzed utilizing flow cytometry. For cell cycle analysis, the propidium iodide (PI) cell cycle detected kits (BD Biosciences) were acquired and used as requested. The percentage of CC cells in G0/G1, S or G2/M phases was detected and results were analyzed by flow cytometer.

### Transwell assay

The 24-well Transwell chambers with 8-mm pore (Corning Incorporated, Big Flats, NY, USA) were implemented to evaluate cell migration and invasion. In detail, CC cells (5 × 10^4^) were placed into the top chamber of Transwell inserts (Corning Incorporated, Corning, NY), which contained Matrigel membrane (BD Biosciences, Franklin Lakes, NJ) for invasion detection (but not for migration assay). The lower chambers were added with complete culture medium (with 10% FBS). Twenty-four hours later, cells invaded or migrated to the bottom were fixed by 4% formaldehyde and stained with crystal violet solution. At length, five fields were selected randomly for counting under the optical microscope (Thermo Fisher).

### Western blot

Cells were lysed in RIPA lysis buffer containing protease inhibitor cocktail and then total protein was collected for protein quantification through Pierce Bicinchoninic acid (BCA) Protein detection kit (Bio-Rad Laboratories, Hercules, CA, USA). Afterwards, 20 µg of protein was subjected to 12% SDS-PAGE, and then proteins were shifted to polyvinylidene difluoride (PVDF) membranes (Bio-Rad Laboratories). After being sealed with 5% BSA, the membranes were processed with primary antibodies at 4 °C overnight. The primary antibodies including anti-GAPDH, anti-cyclin D1, anti-CDK4, anti-caspase-3, anti-cleaved caspase-3, anti-caspase-6, anti-cleaved caspase-6, anti-PARP, anti-RAB10, anti-E6, anti-E7, were all procured from Abcam (Cambridge, MA). After washing in TBST, membranes were probed with HRP-conjugated secondary antibodies (Abcam) for 2 h at room temperature, and then protein signals were analyzed via the enhanced chemiluminescence substrate system (ECL; Santa Cruz Biotechnology, Santa Cruz, CA).

### In vivo study

Ten male BALB/C athymic nude mice (6 weeks of age and weighing 18–20 g) were bought from the National Laboratory Animal Center (Beijing, China) for in vivo study, with the ethical approval from the Institutional Animal Care and Use Committee of Affiliated Hospital of Weifang Medical College. Transfected CC cells (5 × 10^6^) were subcutaneously xenografted into the left flank of mice back, and mice were randomly divided two groups (each group contained 5 mice). Tumor volume was monitored every 4 days. After inoculation for 28 days, mice were sacrificed through cervical dissociation. Thereafter, tumors were dissected and weighed.

### Isolation of nucleus-cytoplasm fractions

The nucleus-cytoplasm fractions were severally isolated from CC cells in accordance with the manual of PARIS™ Kit (Ambion, Austin, TX). Cell samples were centrifuged after lysing in cell fractionation buffer and cell disruption buffer. The target RNAs in fractions were measured by qRT-PCR. GAPDH was used as indicator of cell cytoplasm and U6 served as indicator of cell nucleus.

### RNA immunoprecipitation (RIP)

Magna RIP™ RNA-Binding Protein Immunoprecipitation Kit was used for performing RIP assay, following the user’s guide (Millipore, Bedford, MA). Simply put, after trypsinization and washing by ice-cold PBS for two times, cells were subjected to RIP lysis buffer added with RNase inhibitor and protease inhibitor cocktail. Then, cell lysates were processed with immunoprecipitation with human anti-Ago2 antibody or normal IgG (Millipore; negative control) for one night at 4 °C, with continuous stirring during this process. After that, pre-rinsed magnetic beads (30 μL) were added and then further incubated for half an hour at 37 °C. Finally, the precipitated RNAs were analyzed via qRT-PCR was after being purified.

### Dual-luciferase reporter assay

The full-length LINC00441 or RAB10 3′UTR (untranslated region) fragments covering wild-type and mutant miR-450b-5p binding sites were inserted to the pmirGLO dual-luciferase reporter vectors (Promega, Madison, WI) to obtain pmirGLO-LINC00441-Wt/Mut and pmirGLO-RAB10-Wt/Mut. CC cells with 80% confluent were co-transfected for 48 h with the luciferase vectors including pmirGLO-LINC00441-Wt/Mut and pmirGLO-RAB10-Wt/Mut, and indicated transfection plasmids. Dual-Luciferase Reporter Assay System (Promega, Madison, WI) were employed after 48 h of transfection for analysis of luciferase activity.

### RNA pull down assay

Pierce Magnetic RNA–Protein Pull-Down Kit (Thermo Fisher Scientific, Waltham, MA) was applied for undertaking RNA pull down assay. The RNA extracts from CC cells were collected using RIPA lysis buffer, then incubated with beads and Bio-miR-450b-5p or Bio-NC for 1 h at 4 °C. After washing, qRT-PCR was utilized for determine the RNA enrichment in RNA–protein binding complex.

### Statistical analysis

All experiments contained three biological repeats. Data were analyzed by Student’s t test or one-way ANOVA by use of Graphpad Prism 6 software (La Jolla, CA, USA), with the significant value was set as p < 0.05. Results were displayed as the mean ± standard deviation (S.D.).

## Results

### Silencing LINC00441 inhibits CC cell growth both in vitro and in vivo

**Fig. 1:**
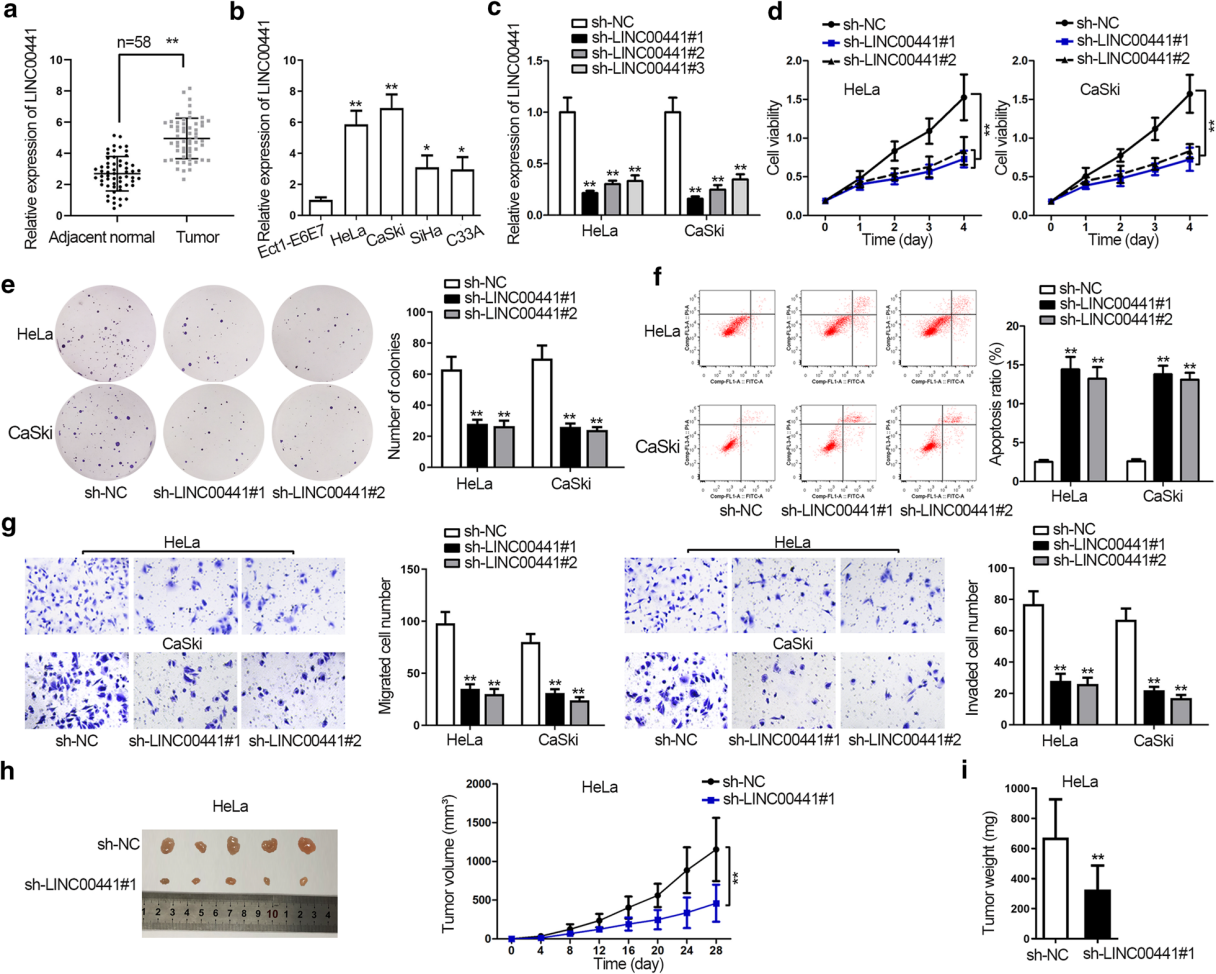
Silencing LINC00441 inhibits CC cell growth both in vitro and in vivo. **a**, **b** LINC00441 expression in CC tissues and cells and control groups was detected by qRT-PCR. **c** LINC00441 knockdown efficiency was examined by qRT-PCR. **d**, **f** Cell viability, proliferation and apoptosis were evaluated by CCK-8, colony formation assay and flow cytometry assay in LINC00441-silenced cells. **g** Transwell assays detected cell migration and invasion in response to LINC00441 downregulation. **h** The pictures of tumors in differently transfected groups were taken and the tumor growth curves of indicated groups were shown. **i** Tumor weight in indicated groups was also measured. ^*^P < 0.05, ^**^P < 0.01

Firstly, we detected that LINC00441 was significantly upregulated in CC tissues compared with control groups (Fig. 1a). Moreover, high expression tendency of LINC00441 in CC cell lines was also validated in Fig. 1b. Besides, HeLa and CaSki cells contained much higher expression of LINC00441 than SiHa and C33A cells among the four CC cell lines (Fig. 1b). Therefore, LINC00441 expression was downregulated by transfecting sh-LINC00441#1/2/3 plasmids in HeLa and CaSki cells, and the knockdown efficiency of sh-LINC00441#1/#2 was more evident (Fig. 1c). Then several loss-of-function experiments was implemented to illustrate the role of LINC00441 in CC. CCK-8 and colony formation assays demonstrated that silencing of LINC00441 suppressed cell proliferation (Fig. 1d, e). While flow cytometry assay examined that LINC00441 depletion accelerated cell apoptosis (Fig. 1f). Besides, flow cytometry analysis of cell cycle distribution disclosed that the percentage of cells was increased in G0/G1, but decreased in S and G2/M phases, upon downregulating LINC00441 in cells (Additional file 1: Figure S1A). The results of western blot assay showed that similar to the changes caused by staurosporine, the expression of CDK4 and cyclin D1 (cell cycle-related proteins) was decreased whereas that of apoptosis-related proteins (cleaved caspase-3, cleaved caspase-6 and cleaved PARP) enhanced in LINC00441 downregulated cells (Additional file 2: Figure S1B). As for cell migration and invasion, transwell assays measured that LINC00441 depletion suppressed the migration and invasion abilities of CC cells (Fig. 1g). As shown in Fig. 1h, i, knockdown of LINC00441 also inhibited the tumor growth of CC, proved by the obviously smaller tumor size and decreased volume and weight compared with control group. Besides, human papillomavirus (HPV) and its oncoproteins (like E6/E7) have been reported to be closely associated with CC progression [23], we therefore investigated whether LINC00441 was affected by HPV proteins in CC cells. As a result, LINC00441 expression kept unchanged in response to E6/E7 overexpression (Additional file 3: Figure S1C). Overall, knockdown of LINC00441 repressed CC cell proliferation, migration, invasion in vitro, as well as inhibited CC tumor growth in vivo.

### LINC00441 sponges with miR-450b-5p in CC cells

**Fig. 2:**
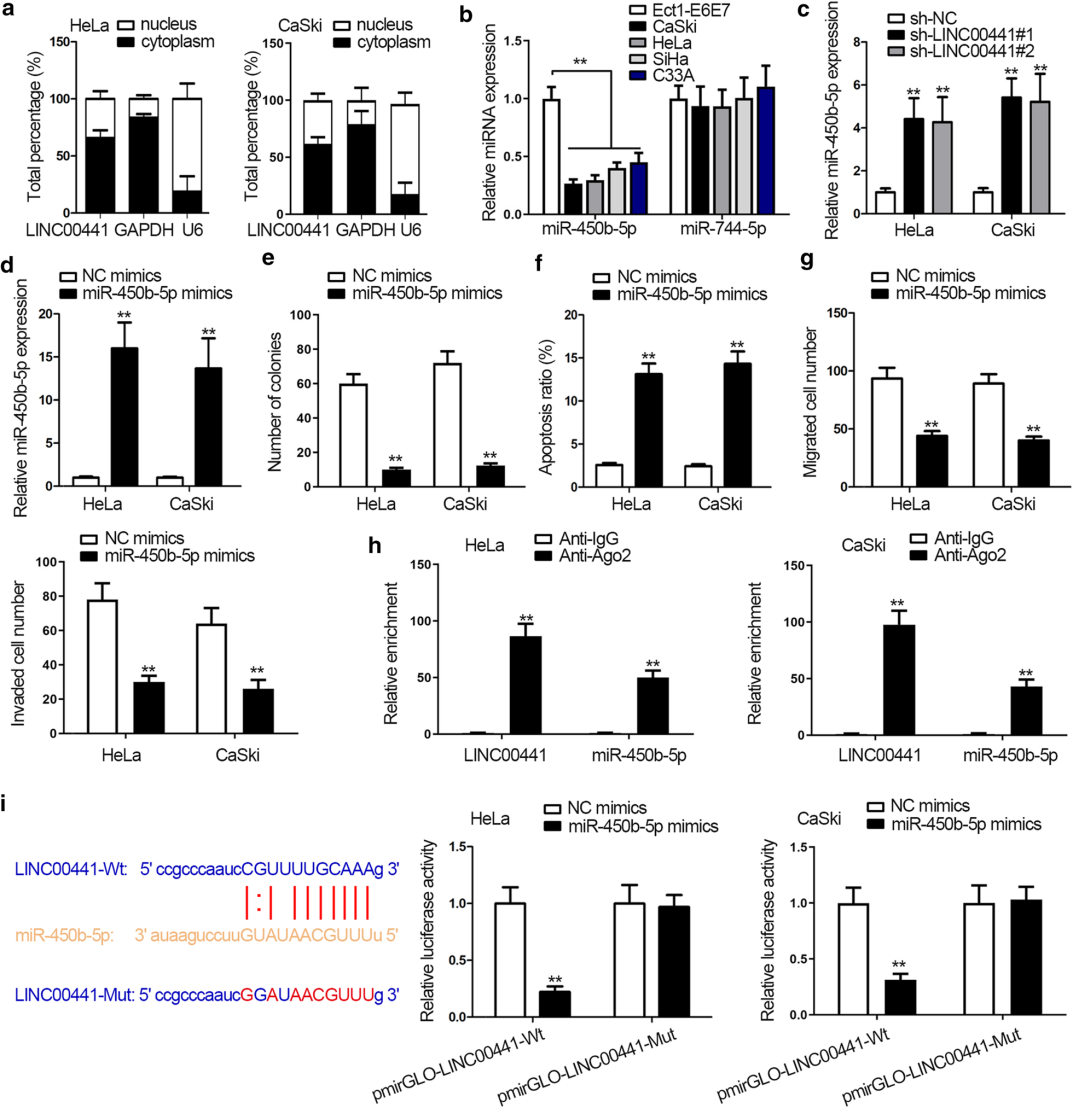
LINC00441 acts as a sponge of miR-450b-5p in CC cells. **a** LINC00441 location in CC cells was determined by isolation of nucleus-cytoplasm fraction. **b** The expression of miRNA candidates in CC cells was assessed by qRT-PCR. **c** MiR-450b-5p expression was measured by qRT-PCR after knockdown of LINC00441. **d** MiR-450b-5p overexpression efficiency was tested by qRT-PCR. **e**–**g** Cell proliferation, apoptosis, migration and invasion were detected through colony formation, flow cytometry and transwell assays after overexpressing miR-450b-5p. **h** RIP assay to the enrichment of the indicated molecules in anti-Ago2 or anti-IgG group. **i** The binding site between miR-450b-5p and LINC00441 was predicted by starBase (left); luciferase reporter assay examined the luciferase activity of pmirGLO-LINC00441-Wt/Mut in miR-450b-5p mimics transfected cells (right). ^**^P < 0.01

To verify our ceRNA network hypothesis, we first detected the location of LINC00441 in cytoplasm and nucleus and found that LINC00441 expression mainly distributed in cytoplasm, indicating the post-transcriptional regulation potential of LINC00441 in CC (Fig. 2a). By retrieving in starBase database (http://starbase.sysu.edu.cn/), we detected there were two miRNAs (miR-450b-5p and miR-744-5p) that possessed binding sites with LINC00441. Next, the relative expression of the two miRNAs was analyzed. qRT-PCR results indicated the expression of miR-450b-5p was downregulated in CC cells while that of miR-744-5p presented no significant change (Fig. 2b). Thus miR-450b-5p was selected in the following researches. Moreover, LINC00441 insufficiency led to an increase in expression of miR-450b-5p (Fig. 2c). To confirm the tumor suppressor role of miR-450b-5p, gain-of-function experiments were applied. Similarly, the efficiency of miR-450b-5p overexpression was detected first with transfecting miR-450b-5p mimics into HeLa and CaSki cells (Fig. 2d). Subsequently, colony formation and flow cytometry assays demonstrated that overexpression of miR-450b-5p led attenuated cell proliferation ability and enhanced cell apoptosis ability (Fig. 2e, f). Afterwards, transwell assays indicated that miR-450b-5p overexpression impaired cell migration and invasion abilities (Fig. 2g). Furthermore, RIP assay examined that miR-450b-5p and LINC00441 both enriched in anti-Ago2 group, indirectly certifying the binding relation between miR-450b-5p and LINC00441 (Fig. 2h). StarBase was used to predict the binding site of miR-450b-5p and LINC00441. And luciferase reporter assay verified the effectiveness of the indicated binding sites due to the sharp decrease in luciferase activity of pmirGLO-LINC00441-Wt in miR-450b-5p mimics transfected cells. And luciferase activity of pmirGLO-LINC00441-Mut wasn’t affected (Fig. 2i). In a word, LINC00441 could sponge miR-450b-5p in CC cells.

### RAB10 is the target gene of miR-450b-5p

**Fig. 3:**
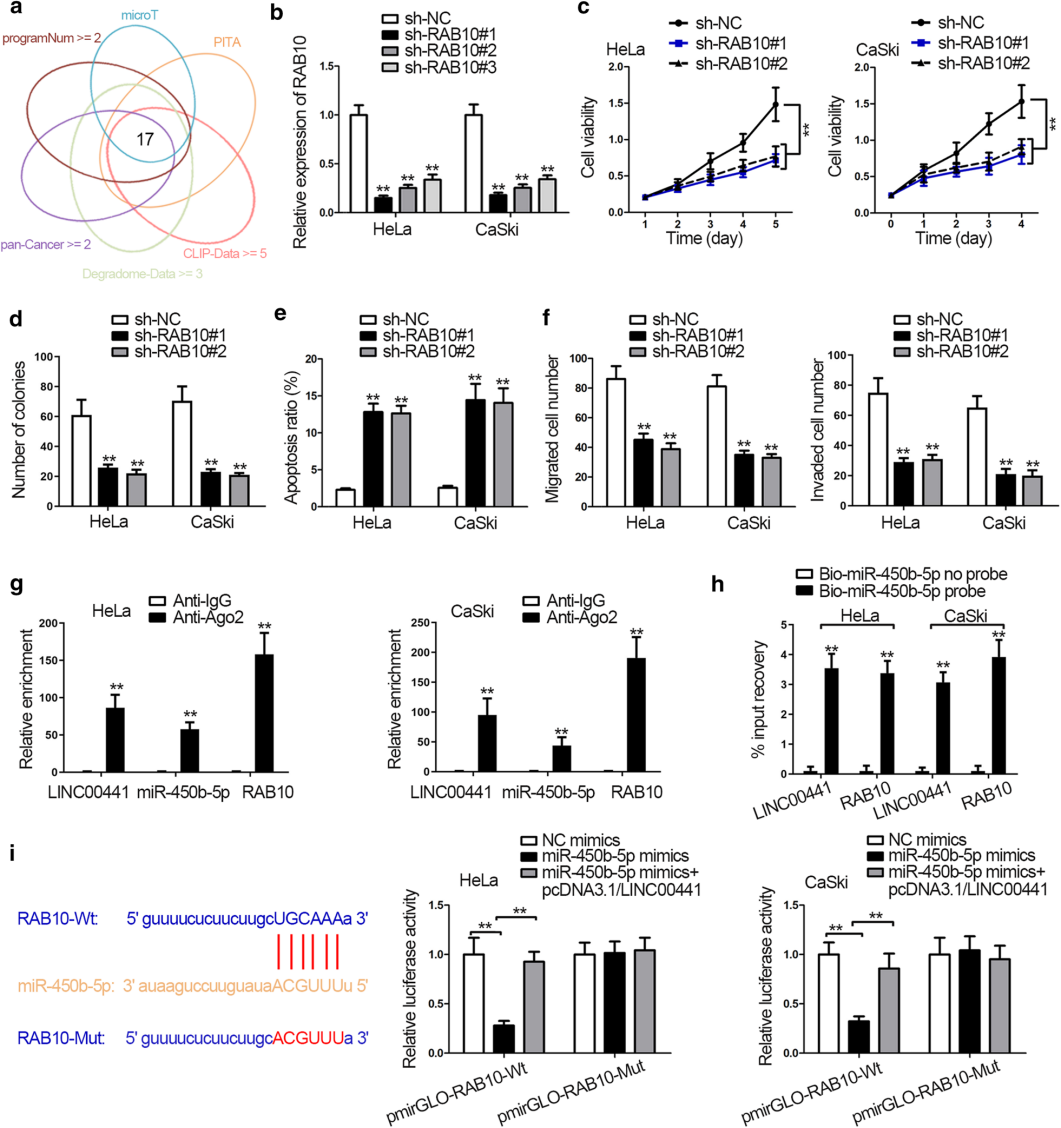
RAB10 is the target gene of miR-450b-5p. **a** MRNA candidates were selected by starBase. **b** RAB10 knockdown efficiency was examined by qRT-PCR. **c**–**e** Cell viability, proliferation and apoptosis were tested by CCK-8, colony formation assay and flow cytometry assay in response to RAB10 silencing. **f** Transwell assays detected cell migration and invasion when downregulating RAB10. **g** RIP assay measured enrichment of the indicated molecules in anti-Ago2 or anti-IgG group. **h** RNA pull down assay examined the PCR products in Bio-miR-450b-5p no probe or Bio-miR-450b-5p probe group. **i** StarBase predicted the binding site of RAB10 and miR-450b-5p (left). Luciferase reporter assay examined luciferase activity of pmirGLO-RAB10-Wt/Mut in miR-450b-5p mimics transfected cells (right). ^**^P < 0.01

To find out the possible downstream mRNA of miR-450b-5p, 17 mRNAs that might bind with miR-450b-5p were obtained using starBase (Fig. 3a). The expression of above mRNAs in CC cells was detected. EIF5 and RAB10 were significantly upregulated in cells (Additional file 2: Figure S2A). For further filtration, the expression of EIF5 and RAB10 was tested in sh-LINC00441 transfected cells, and only RAB10 expression was suppressed (Additional file 2: Figure S2B). Therefore, RAB10 was determined as the researching object. Moreover, the role of RAB10 in CC was also explained through loss-of-function experiments. RAB10 knockdown efficiency was examined by qRT-PCR and western blot assays (Fig. 3b and Additional file 2: S2C). Results from CCK-8, colony formation, and flow cytometry assays attested that cell proliferation was decreased while apoptosis was increased upon RAB10 silencing (Fig. 3c–e). Additionally, transwell assay tested that RAB10 knockdown suppressed cell migration and invasion (Fig. 3f). The following RIP assay proved that LINC00441, miR-450b-5p and RAB10 coexisted in RNA-induced silencing complex (RISC) (Fig. 3g). Moreover, RNA pull down assay confirmed the great enrichment of LINC00441 and RAB10 in Bio-miR-450b-5p group, validating the interactions among LINC00441, miR-450b-5p and RAB10 in CC cells (Fig. 3h). In a similar way, luciferase reporter assay verified the putative binding sites between miR-450b-5p and RAB10 obtained from starBase. Overexpressing miR-450b-5p decreased the luciferase activity of pmirGLO-RAB10-Wt, and LINC00441 upregulation could rescue this inhibitory role (Fig. 3i). In a general, RAB10 was the target gene of miR-450b-5p, and LINC00441 could regulate the expression of RAB10 via sponging miR-450b-5p in CC cells.

### Knockdown of miR-450b-5p or overexpression of RAB10 reverses the effects of silencing LINC00441 on CC cell functions

**Fig. 4:**
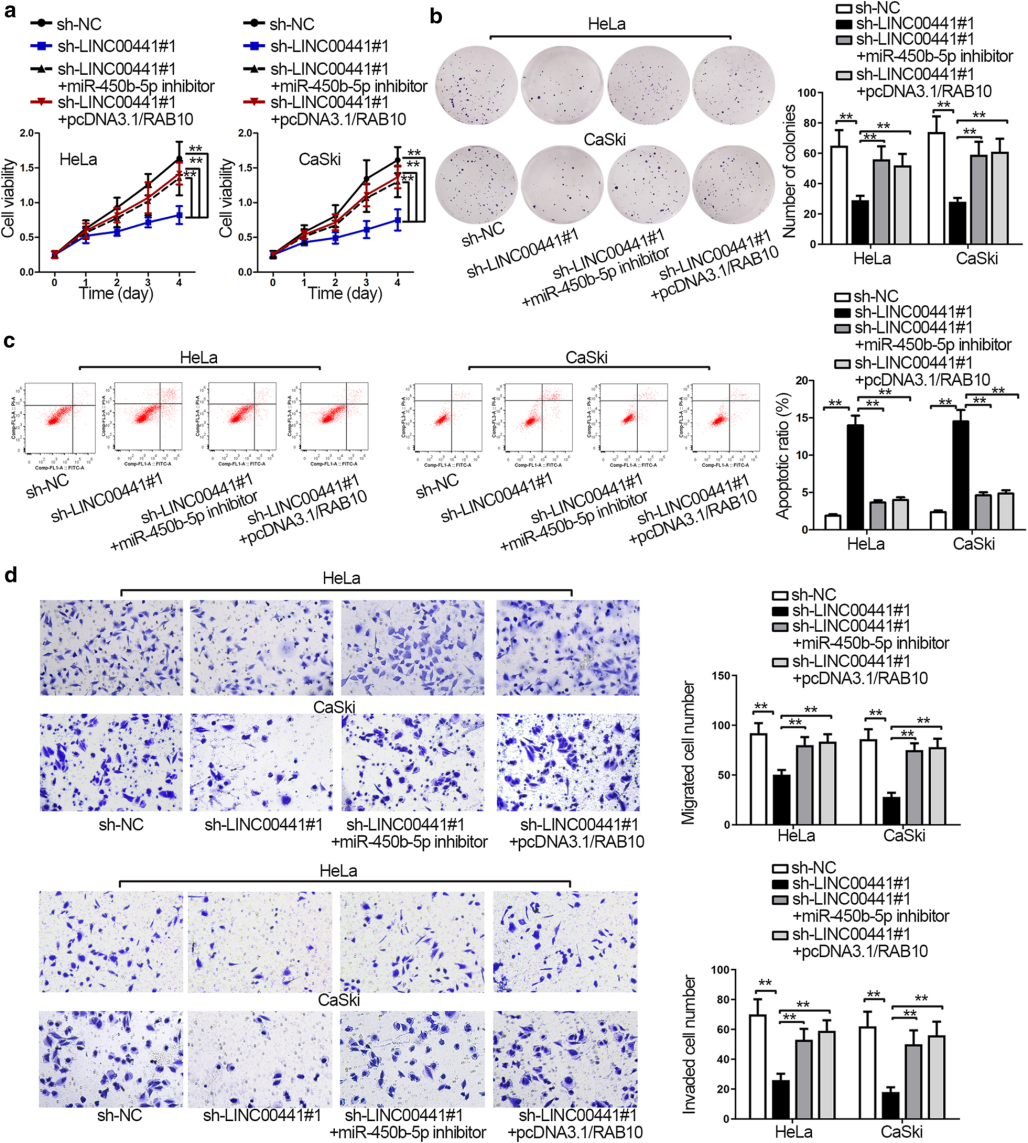
Knockdown of miR-450b-5p or overexpression of RAB10 reverses the effects of silencing LINC00441 on CC. **a**–**c** CCK-8 assay, colony formation assay and flow cytometry analysis demonstrated cell viability, proliferation and apoptosis in different groups. **d** Cell migration and invasion in transfected cells were evaluated by transwell assays. ^**^P < 0.01

Finally, to confirm whether LINC00441 promoted CC progression by modulating the miR-450b-5p/RAB10 axis, we performed rescue experiments. From CCK-8 and colony formation assay, we observed both miR-450b-5p inhibitor and pcDNA3.1/RAB10 groups could reverse the hindering function caused by LINC00441 knockdown on cell viability and proliferation (Fig. 4a, b). Flow cytometry assay demonstrated that miR-450b-5p knockdown or RAB10 upregulation offset the stimulating effects of LINC00441 deficiency on cell apoptosis (Fig. 4c). Meanwhile, flow cytometry analysis also illustrated that the cell cycle arrest induced by LINC00441 knockdown was rescued by miR-450b-5p inhibitor or pcDNA3.1/RAB10 (Additional file 3: Figure S3A). Additionally, the results of western blot assays displayed that miR-450b-5p suppression or RAB10 augmentation neutralized the effects of LINC00441 depletion on expression of cell cycle- and apoptosis- associated proteins (Additional file 3: Figure S3B). Moreover, transwell assays detected that miR-450b-5p downregulation or RAB10 overexpression remedied the inhibited migration and invasion in sh-LINC00441#1-transfected cells (Fig. 4d). To sum up, LINC00441 promoted CC proliferation, migration and invasion by targeting miR-450b-5p/RAB10 axis.

## Discussion

Thousands of lncRNAs have been identified during the progression of various diseases including cancers, thanks to the human transcriptome sequencing technologies [24]. An increasing number of lncRNAs are identified to play a vital role in tumorigenesis and progression. Except for the lncRNAs that have been studied in CC, such as HOXD-AS1 [25], TUG1 [26], MEG3 [27] and OGFRP1 [28], there is still a long way to go to identify and characterize new lncRNAs for control of CC initiation and progression. More importantly, LINC00441 was upregulated in CC tissue samples compared with normal tissues. Further, qRT-PCR analysis validated LINC00441 presented high expression tendency in CC cell lines. The following loss-of-function experiments proved that knockdown of LINC00441 repressed CC cell proliferation, migration and invasion and induced cell cycle arrest, but accelerated cell apoptosis in vitro. Meanwhile, LINC00441 depletion suppressed tumor growth in vivo, which consistent with previous findings. LINC00441 was an oncogenic gene in gastric cancer and hepatocellular carcinoma [12, 13]. And our study was the first to investigate the role of LINC00441 in CC.

Many studies have demonstrated that lncRNAs could function as a ceRNA to modulate target mRNA expression by sponging miRNA in various cancers including CC. For instance, lncRNA TUG1 sequesteres miR-204-5p and drives osteoblast differentiation via elevating expression of Runx2 in aortic valve calcification [29]. SP1-mediated lncRNA POU3F3 contributes to CC progression through miR-127-5p/FOXD1 axis [30]. LncRNA DLG1-AS1 stimulates CC cell proliferation via competitively binding with miR-107 to upregulate ZHX1 expression [31]. In our study, miR-450b-5p had binding sites with LINC00441. The expression of miR-450b-5p was significantly downregulated in CC cells. The published papers have reported that miR-450b-5p functions as a tumor suppressor in various cancers including colorectal cancer, hepatocellular carcinoma and lung adenocarcinoma, etc. [16, 32, 33]. In the presented study, we confirmed that LINC00441 was the sponge of miR-450b-5p. Furthermore, the upregulated mRNA RAB10 in CC cells interested us. Previous studies have demonstrated that RAB10 is an oncogenic gene in esophageal squamous cell carcinoma, osteosarcoma and hepatocellular carcinoma [34–37]. Finally, rescue experiments detected that RAB10 overexpression or miR-450b-5p knockdown could reverse LINC00441 depletion-mediated function on cell proliferation, apoptosis, migration, invasion and cell cycle in vitro as well as tumor growth in vivo, indicating that LINC00441 promoted CC progression by sponging miR-450b-5p to upregulate RAB10 expression.

## Conclusion

**Fig. 5:**
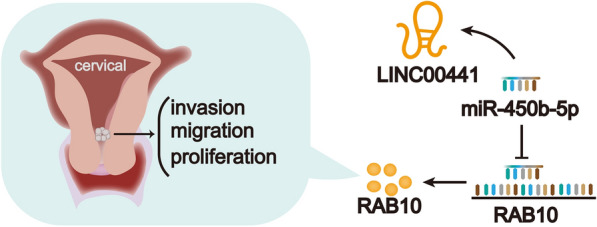
The graphical diagram of LINC00441/miR-450b-5p/RAB10 axis in CC

The study demonstrated LINC00441 promotes cell proliferation, migration, invasion and tumor growth in CC by sponging miR-450b-5p to upregulate RAB10 expression, as illustrated by Fig. 5.

## Supplementary information

**Additional file 1: Figure S1**. **A.** Flow cytometry analyzed cell cycle distribution in response to LINC00441 silencing. **B.** Western blot assay measured cell cycle- and apoptosis-related protein expression in Hela or CaSki cells transfected with sh-NC or sh-LINC00441#1/2, or treated with Staurosporine. **C.** Western blot assay measured overexpression efficiency of E6/E7 (left); qRT-PCR measured the expression of LINC00441 when upregulating E6/E7 (right). ^**^P < 0.01.

**Additional file 2: Figure S2. A.** qRT-PCR measured the expression of mRNA candidates in CC cells and normal cervical cells. **B.** qRT-PCR measured the expression of EIF5 and RAB10 in sh-LINC00441#1/2/3 transfected CC cells. **C.** Western blot analysis measured the knockdown efficiency of RAB10. ^**^P < 0.01.

**Additional file 3: Figure S3 A.** Flow cytometry analyzed cell cycle distribution in transfected cells. **B.** Western blot assay measured cell cycle- and apoptosis-related protein expression in indicated CC cells. ^**^P < 0.01.

## Data Availability

Not applicable.

## Abbreviations

ANOVA: Analysis of Variance
ANRIL: Antisense non-coding RNA in the INK4 locus
ATCC: American Type Culture Collection
CC: Cervical cancer
CCK-8: Cell-Counting Kit 8
cDNA: Complementary DNA
DLG1-AS1: Discs large MAGUK scaffold protein 1 antisense RNA 1
DMEM: Dulbecco’s Modified Eagle Medium
ECL: Enhanced chemiluminescence
EGOT: Eosinophil granule ontogeny transcript
FBS: Fetal bovine serum
FOXD1: Forkhead box D1
GAS5: Growth arrest specific 5
HCC: Hepatocellular carcinoma
HMGA2: High mobility group AT-hook 2
HOXD-AS1: Homeobox D cluster antisense RNA 1
KIF26B: Kinesin family member 26B
lncRNAs: Long non-coding RNAs
MEG3: Maternally expressed 3
miRNAs: microRNAs
MMC: Mitomycin C
mRNAs: Messenger RNAs
MTDH: Metadherin
NEAT1: Nuclear paraspeckle assembly transcript 1
OGFRP1: Opioid growth factor receptor pseudogene 1
PAX8: Paired box 8
POU3F3: POU class 3 homeobox 3
PVDF: Polyvinylidene Fluoride
qRT-PCR: Quantitative real-time polymerase chain reaction
RAB10: Ras-related protein Rab-10
RIP: RNA immunoprecipitation
RIPA: Radioimmunoprecipitation assay
RISC: RNA induced silencing complex
Runx2: RUNX family transcription factor 2
S.D.: Standard deviation
SNHG1: Small nucleolar RNA host gene 1
SOCS3: Suppressor of cytokine signaling 3
TBST: Tris Buffered Saline Tween
TUG1: Taurine up-regulated 1
ZHX1: Zinc fingers and homeoboxes 1
