# Supplementary figures and images for: LINC00441 promotes cervical cancer progression by modulating miR-450b-5p/RAB10 axis
Source: Cancer Cell Int. 2020 Aug 4;20:368. doi: 10.1186/s12935-020-01400-x (PMC7409438; doi:10.1186/s12935-020-01400-x)

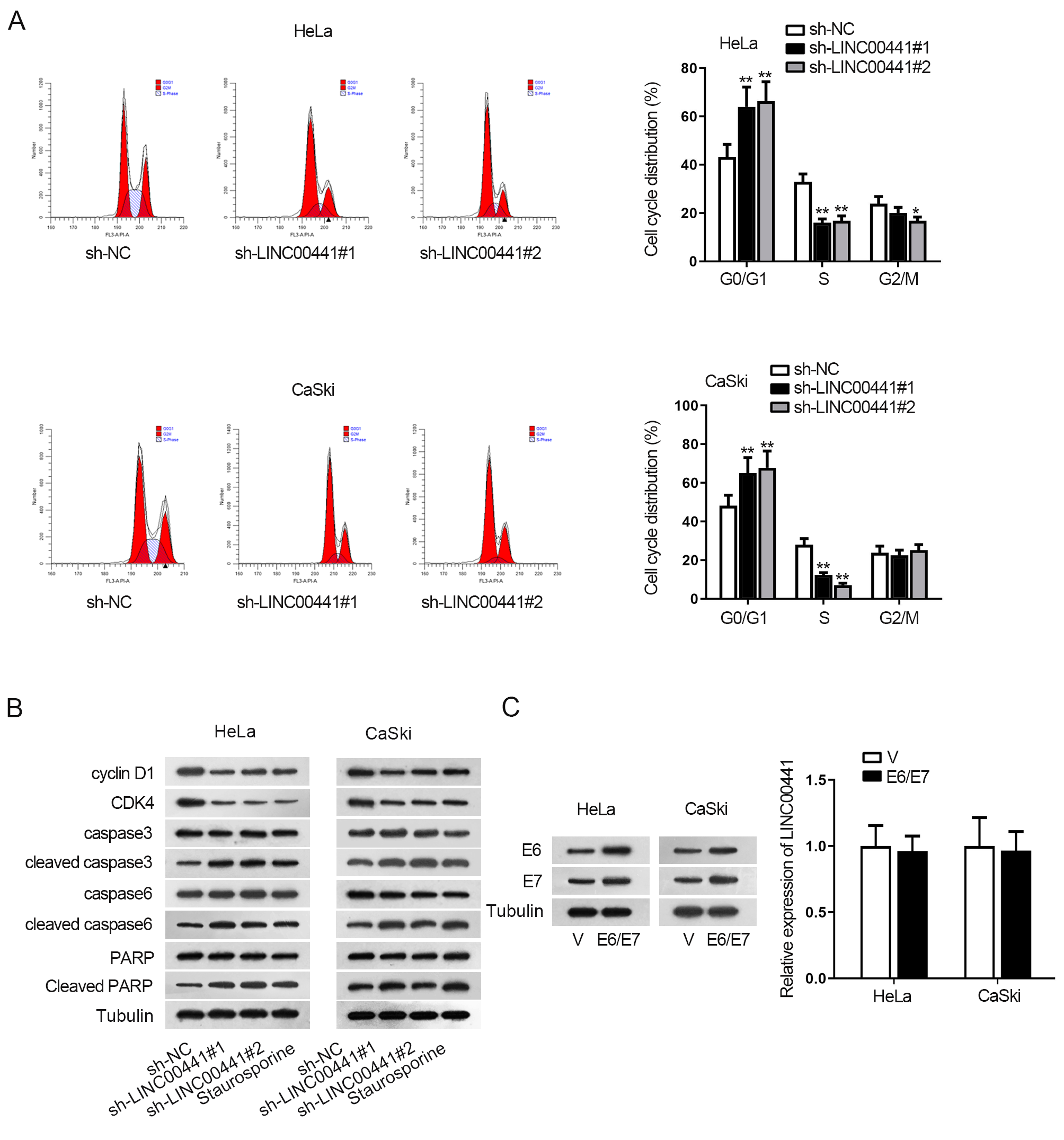

Supplement: Supplementary file 1 — Additional file 1: Figure S1. A. Flow cytometry analyzed cell cycle distribution in response to LINC00441 silencing. B. Western blot assay measured cell cycle- and apoptosis-related protein expression in Hela or CaSki cells transfected with sh-NC or sh-LINC00441#1/2, or treated with Staurosporine. C. Western blot assay measured overexpression efficiency of E6/E7 (left); qRT-PCR measured the expression of LINC00441 when upregulating E6/E7 (right). **P < 0.01. [file 12935_2020_1400_MOESM1_ESM.tif]

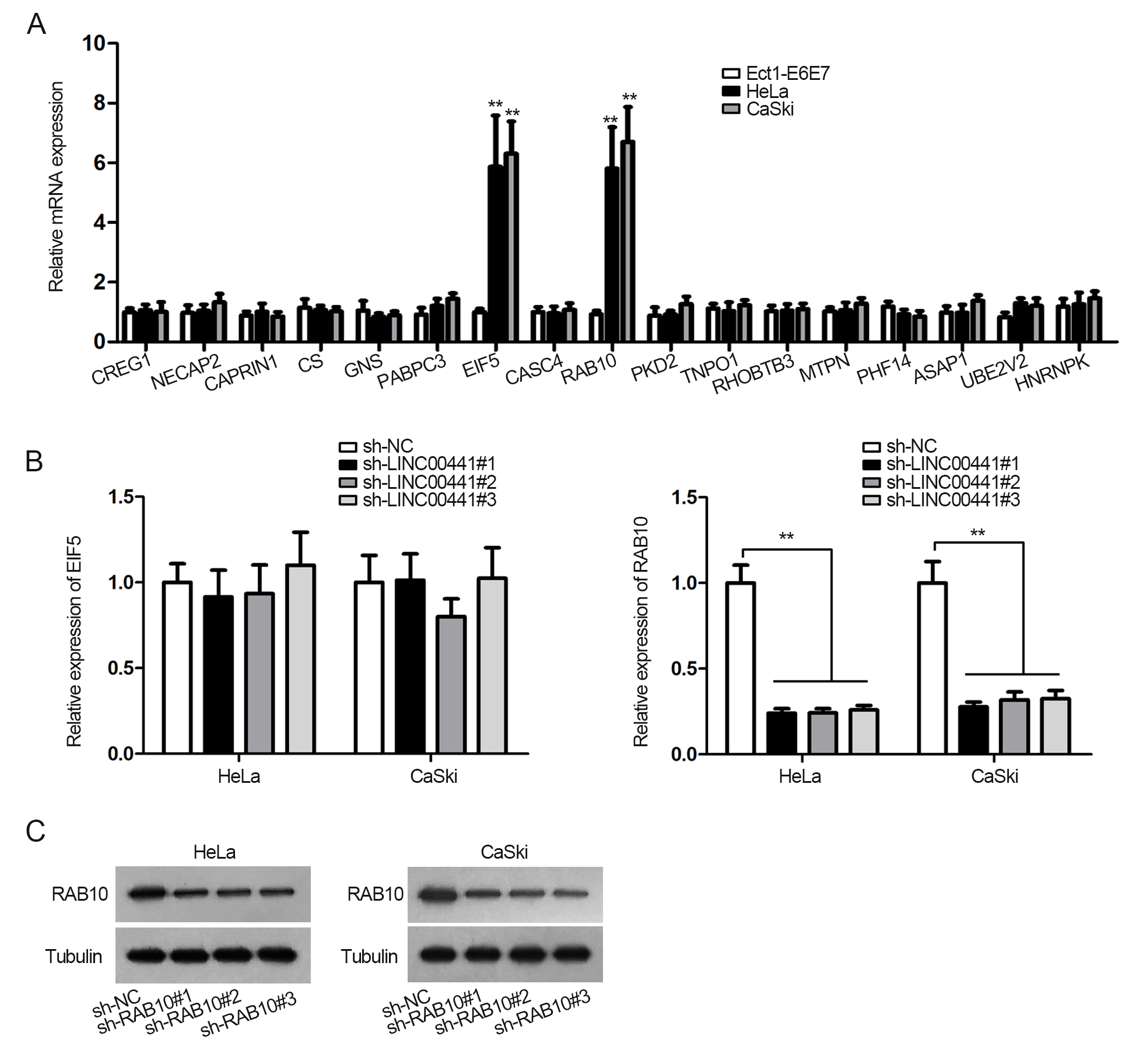

Supplement: Supplementary file 2 — Additional file 2: Figure S2. A. qRT-PCR measured the expression of mRNA candidates in CC cells and normal cervical cells. B. qRT-PCR measured the expression of EIF5 and RAB10 in sh-LINC00441#1/2/3 transfected CC cells. C. Western blot analysis measured the knockdown efficiency of RAB10. **P < 0.01. [file 12935_2020_1400_MOESM2_ESM.tif]

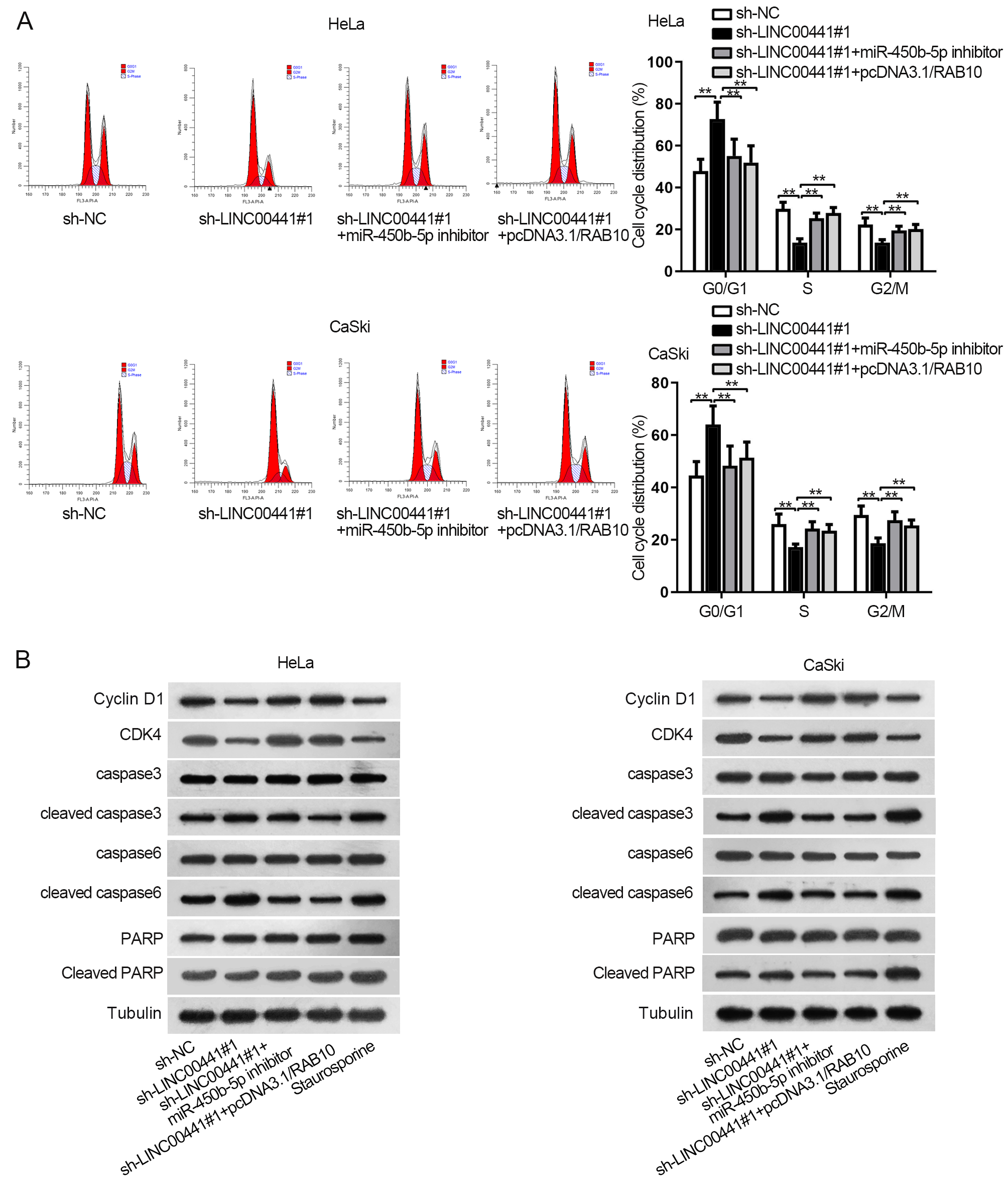

Supplement: Supplementary file 3 — Additional file 3: Figure S3 A. Flow cytometry analyzed cell cycle distribution in transfected cells. B. Western blot assay measured cell cycle- and apoptosis-related protein expression in indicated CC cells. **P < 0.01. [file 12935_2020_1400_MOESM3_ESM.tif]
